# Supplementary material for: Antimicrobial Resistance Profiles of Escherichia coli from Diarrheic Weaned Piglets after the Ban on Antibiotic Growth Promoters in Feed
Source: Antibiotics (Basel). 2020 Oct 29;9(11):755. doi: 10.3390/antibiotics9110755 (PMC7693254; doi:10.3390/antibiotics9110755)
Supplement: Supplementary file 1 [file antibiotics-09-00755-s001.pdf]

antibiotics

MDPI

Table S1. Antimicrobial-resistant pathogenic *Escherichia coli* isolates (%) from weaned piglets with diarrhea in northern, middle, and southern Korean farms before and after the ban on AGPs in feed.

Anti-microbial agents[1]	Northern farms (Gangwon, Gyeonggi, and Incheon)			Middle farms (Chungbuk, and Chungnam)			Southern farms (Chonbuk, Chonnam, Gyeonggi, and Gyeongnam)		
	Before (2007-2011) (n=61)	After (2012-2017) (n=74)	Total (2007-2017) (n=135)	Before (2007-2011) (n=74)	After (2012 -2017) (n=216)	Total (2007-2017) (n=290)	Before (2007-2011) (n=83)	After (2012-2017) (n=182)	Total (2007-2017) (n=265)
**GM**	45 (73.8)	38 (51.4)	83 (61.5)	50 (67.6)	78 (36.1)	128 (44.1)	55 (66.3)	68 (37.4)	123 (46.4)
**S**	48 (78.7)	62 (83.8)	110 (81.5)	64 (86.5)	185 (85.6)	249 (85.9)	72 (86.7)	158 (86.8)	230 (86.8)
**N**	52 (85.2)	43 (58.1)	95 (70.4)	64 (86.5)	125 (57.9)	189 (65.2)	69 (83.1)	105 (57.7)	174 (65.7)
**CF**	26 (42.6)	52 (70.3)	78 (57.8)	40 (54.1)	153 (70.8)	193 (66.6)	46 (55.4)	123 (67.6)	169 (63.8)
**CZ**	12 (19.7)	17 (23.0)	29 (21.5)	14 (18.9)	50 (23.1)	64 (22.1)	15 (18.1)	40 (22.0)	55 (20.8)
**FEP**	0 (0.0)	2 (2.7)	2 (1.5)	0 (0.0)	5 (2.3)	5 (1.7)	0 (0.0)	3 (1.6)	3 (1.1)
**FOX**	10 (16.4)	11 (14.9)	21 (15.6)	10 (13.5)	33 (15.3)	43 (14.8)	13 (15.7)	17 (9.3)	30 (11.3)
**NA**	46 (75.4)	55 (74.3)	101 (74.8)	57 (77.0)	146 (67.6)	203 (70.0)	60 (72.3)	122 (67.0)	182 (68.7)
**CIP**	33 (54.1)	36 (48.6)	69 (51.1)	39 (52.7)	86 (39.8)	125 (43.1)	36 (43.4)	65 (35.7)	101 (38.1)
**NOR**	29 (47.5)	34 (45.9)	63 (46.7)	38 (51.4)	81 (37.5)	119 (41.0)	35 (42.2)	61 (33.5)	96 (36.2)
**AM**	50 (82.0)	64 (86.5)	114 (84.4)	63 (85.1)	180 (83.3)	243 (83.8)	76 (91.6)	153 (84.1)	229 (86.4)
**AMC**	20 (32.8)	31 (41.9)	51 (37.8)	32 (43.2)	80 (37.0)	112 (38.6)	37 (44.6)	56 (30.8)	93 (35.1)
**SXT**	37 (60.7)	52 (70.3)	89 (65.9)	46 (62.2)	126 (58.3)	172 (59.3)	50 (60.2)	109 (59.9)	159 (60.0)
**C**	49 (80.3)	70 (94.6)	119 (88.1)	72 (97.3)	185 (85.6)	257 (88.6)	73 (88.0)	154 (84.6)	227 (85.7)
**CL**	2 (3.3)	7 (9.5)	9 (6.7)	7 (9.5)	27 (12.5)	34 (11.7)	7 (8.4)	18 (9.9)	25 (9.4)
**TE**	52 (85.2)	63 (85.1)	115 (85.2)	70 (94.6)	174 (80.6)	244 (84.1)	79 (95.2)	160 (87.9)	239 (90.2)

[1] GM: gentamicin, S: streptomycin, N: neomycin, CF: cephalothin, CZ: cefazolin, FEP: cefepime, FOX: cefoxitin, NA: nalidixic acid, CIP: ciprofloxacin, NOR: norfloxacin, AM: ampicillin, AMC: amoxicillin/clavulanic acid, SXT: trimethoprim/sulfamethoxazole, C: chloramphenicol, CL: colistin, TE: tetracycline

Table S2. Multidrug-resistant pathogenic *Escherichia coli* isolates (%) from weaned piglets with diarrhea in northern, middle, and southern Korean farms before and after the ban on AGPs in feed.

Anti-microbial subclass[1]	Northern farms (Gangwon, Gyeonggi, and Incheon)			Middle farms (Chungbuk, and Chungnam)			Southern farms (Chonbuk, Chonnam, Gyeonggi, and Gyeongnam)		
	Before (2007-2011) (n=61)	After (2012-2017) (n=74)	Total (2007-2017) (n=135)	Before (2007-2011) (n=74)	After (2012-2017) (n=216)	Total (2007-2017) (n=290)	Before (2007-2011) (n=83)	After (2012-2017) (n=182)	Total (2007-2017) (n=265)
**0 subclass**	4 (6.6)	0 (0.0)	4 (3.0)	0 (0.0)	0 (0.0)	0 (0.0)	0 (0.0)	0 (0.0)	0 (0.0)
**1 subclass**	2 (3.3)	0 (0.0)	2 (1.5)	0 (1.4)	3 (1.4)	3 (1.0)	0 (0.0)	3 (1.6)	3 (1.1)
**2 subclasses**	1 (1.6)	0 (0.0)	1 (0.7)	1 (1.4)	3 (1.4)	4 (1.4)	3 (3.6)	7 (3.8)	10 (3.8)
**3 subclasses**	1 (1.6)	3 (4.1)	4 (3.0)	1 (1.4)	11 (5.1)	12 (4.1)	1 (1.2)	5 (2.7)	6 (2.3)
**4 subclasses**	4 (6.6)	3 (4.1)	7 (5.2)	4 (5.4)	23 (10.6)	27 (9.3)	6 (7.2)	18 (9.9)	24 (9.1)
**5 subclasses**	8 (13.1)	9 (12.2)	17 (12.6)	11 (14.9)	33 (15.3)	44 (15.2)	12 (14.5)	30 (16.5)	42 (15.8)
**6 subclasses**	8 (13.1)	12 (16.2)	20 (14.8)	16 (21.6)	32 (14.8)	48 (16.6)	16 (19.3)	26 (14.3)	42 (15.8)
**7 subclasses**	15 (24.6)	21 (28.4)	36 (26.7)	17 (23.0)	45 (20.8)	62 (21.4)	18 (21.7)	33 (18.1)	51 (19.2)
**8 subclasses**	5 (8.2)	13 (17.6)	18 (13.3)	8 (10.8)	26 (12.0)	34 (11.7)	10 (12.0)	37 (20.3)	47 (17.7)
**9 subclasses**	4 (6.6)	7 (9.5)	11 (8.1)	7 (9.5)	17 (7.9)	24 (8.3)	8 (9.6)	13 (7.1)	21 (7.9)
**10 subclasses**	9 (14.8)	4 (5.4)	13 (9.6)	8 (10.8)	22 (10.2)	30 (10.3)	9 (10.8)	9 (4.9)	18 (6.8)
**11 subclasses**	0 (0.0)	2 (2.7)	2 (1.5)	1 (1.4)	1 (0.5)	2 (0.7)	0 (0.0)	1 (0.5)	1 (0.4)
**Multi-resistant (≥ 3subclasses)**	54 (88.5)	74 (100.0)	128 (94.8)	78 (98.6)	210 (97.2)	283 (97.6)	80 (96.4)	172 (94.5)	252 (95.1)

[1] Antimicrobial subclass are defined by the Clinical and Laboratory Standards Institute.

Table S3. Antimicrobial resistance genes (%) of pathogenic *Escherichia coli* from weaned piglets with diarrhea in northern, middle, and southern Korean farms before and after the ban on AGPs in feed.

Anti-microbial agents	Northern farms (Gangwon, Gyeonggi, and Incheon)			Middle farms (Chungbuk, and Chungnam)			Southern farms (Chonbuk, Chonnam, Gyeonggi, and Gyeongnam)		
	Before (2007-2011) (n=61)	After (2012-2017) (n=74)	Total (2007-2017) (n=135)	Before (2007-2011) (n=74)	After (2012 -2017) (n=216)	Total (2007-2017) (n=290)	Before (2007-2011) (n=83)	After (2012-2017) (n=182)	Total (2007-2017) (n=265)
blaTEM	36 (59.0)	38 (51.4)	74 (54.8)	55 (74.3)	102 (47.2)	157 (54.1)	60 (72.3)	92 (50.5)	152 (57.4)
blaSHV	2 (3.3)	5 (6.8)	7 (5.2)	1 (1.4)	16 (7.4)	17 (5.9)	2 (2.4)	8 (4.4)	10 (3.8)
blaOXA	7 (11.5)	5 (6.8)	12 (8.9)	8 (10.8)	14 (6.5)	22 (7.6)	9 (10.8)	27 (14.8)	36(13.6)
blaCTX-M group 1	0 (0.0)	1 (1.4)	1 (0.7)	4 (5.4)	6 (2.8)	10 (3.4)	0 (0.0)	2 (1.1)	2 (0.8)
blaCTX-M group 2	0 (0.0)	0 (0.0)	0 (0.0)	0 (0.0)	3 (1.4)	3 (1.0)	0 (0.0)	5 (2.7)	5 (1.9)
blaCTX-M group 9	0 (0.0)	1 (1.4)	1 (0.7)	4 (5.4)	9 (4.2)	13 (4.5)	0 (0.0)	4 (2.2)	4 (1.5)
mcr-1	0 (0.0)	1 (1.4)	1 (0.7)	0 (0.0)	3 (1.4)	3 (1.0)	1 (1.2)	3 (1.6)	4 (1.5)
mcr-2	0 (0.0)	0 (0.0)	0 (0.0)	0 (0.0)	0 (0.0)	0 (0.0)	0 (0.0)	0 (0.0)	0 (0.0)
mcr-3	0 (0.0)	1 (1.4)	1 (0.7)	2 (2.7)	3 (1.4)	5 (1.7)	1 (1.2)	1 (0.5)	2 (0.8)
AmpC	31 (50.8)	52 (70.3)	83 (61.5)	28 (37.8)	145 (67.1)	173 (59.7)	38 (45.8)	120 (65.9)	158 (59.6)
tetA	44 (72.1)	56 (75.7)	100 (74.1)	59 (79.7)	144 (66.7)	203 (70.0)	69 (83.1)	119 (65.4)	188 (70.9)

Table S4. Primers for detection of antimicrobial resistance genes.

**Resistance gene**	**Sequence (5′ to 3′)[1]**	**Tm[2]**	**Product length**	**Reference**
mcr-1 FWD<br>*mcr*-1 REV	CGGTCAGTCCGTTTGTTC<br>CTTGGTCGGTCTGTAGGG	55	309	[5]
mcr-2 FWD<br>*mcr*-2 REV	TGTTGCTTGTGCCGATTGGA<br>AGATGGTATTGTTGGTTGCTG	65	567	
mcr-3 FWD<br>*mcr*-3 REV	TTGGCACTGTATTTTGCATTT<br>TTAACGAAATTGGCTGGAACA	55	542	
tetA FWD<br>*tetA* REV	GCGCGATCTGGTTCACTCG<br>AGTCGACAGYRGCGCCGGC	61	164	[32]
AmpC FWD<br>*AmpC* REV	AATGGGTTTTCTACGGTCTG<br>GGGCAGCAAATGTGGAGCAA	55	191	[33]
blaTEM FWD<br>*blaTEM* REV	CATTTCCGTGTCGCCCTTAATTC<br>CGTTCATCCATAGTTGCCTGAC	60	800	[34]
blaSHV FWD<br>*blaSHV* REV	AGCCGCTTGAGCAAATTAAAC<br>ATCCCGCAGATAAATCACCAC	60	713	
blaOXA FWD<br>*blaOXA* REV	GGCACCAGATTCAACTTTCAAG<br>GACCCCAAGTTTCCTGTAAGTG	60	564	
blaCTX-M group 1 FWD<br>*blaCTX-M* group 1 REV	TTAGGAARTGTGCCGTGYA<br>CGATATCGTTGGTGGTRCCAT	60	688	
blaCTX-M group 2 FWD<br>*blaCTX-M* group 2 REV	CGTTAACGGCACGATGAC<br>CGATATCGTTGGTGGTRCCAT	60	404	
blaCTX-M group 9 FWD<br>*blaCTX-M* group 9 REV	TCAAGCCTGCCGATCTGGT<br>TGATTCTCGCCGCTGAAG	60	561	

[1] Y = T or C; R = A or G

[2] Annealing temperatures (^{o}C)
